# Perceptions, motivations, needs and experiences of geriatricians with regard to research and research consortia

**DOI:** 10.1007/s40520-026-03426-z

**Published:** 2026-06-29

**Authors:** Julia Minnema, Isabelle S. Moens, Yvonne M. Drewes, Floor J. A. van Deudekom, Jacobijn Gussekloo, Mirella M. N. Minkman, Simon P. Mooijaart, Sarah H. M. Robben, Hanna C. Willems, Hugo P. M. van der Kuy, Francesco Mattace-Raso, Harmke A. Polinder-Bos, Miriam C. Faes

**Affiliations:** 1 Division of Geriatric Medicine, Department of Internal Medicine, Erasmus MC, University Medical Center, Rotterdam, The Netherlands; 2 Department of Hospital Pharmacy, Erasmus MC, University Medical Center, Rotterdam, The Netherlands; 3 Department of Internal Medicine, Section Gerontology and Geriatrics, LUMC, Leiden, The Netherlands; 4 LUMC Center for Medicine for Older people, Leiden University Medical Center, Leiden, The Netherlands; 5 Department of Geriatrics, OLVG Hospital, Amsterdam, The Netherlands; 6 Department of Public Health and Primary Care, Leiden University Medical Center, Leiden, The Netherlands; 7 Tilburg University TIAS, Tilburg, The Netherlands; 8 Center of Excellence for care and support, Vilans, Utrecht, The Netherlands; 9 Department of Geriatric Medicine, Elisabeth-TweeSteden Hospital, Tilburg, The Netherlands; 10 Department of Internal Medicine and Geriatrics, Amsterdam University Medical Center location AMC, Amsterdam, The Netherlands; 11 Department of Geriatrics, Amphia Hospital, Breda, The Netherlands

**Keywords:** Research, Consortia, Collaboration, Geriatrics

## Abstract

**Background:**

With the aging population, there is a growing need for research in geriatrics. Currently, participation of geriatricians in research is behind that of other specialties.

**Aim:**

We aim to investigate the perceptions, motivations, needs and experiences of geriatricians with regard to research and their willingness to contribute to research consortia in the Netherlands.

**Methods:**

The study was a sequential explanatory mixed-methods study. Between March 2024 and May 2024, all Dutch geriatricians were invited to participate in an online survey, then semi-structured interviews were conducted. An inductive thematic analysis was used to identify, analyse, and report patterns in the qualitative data.

**Results:**

171 geriatricians (80.6% female) participated in the survey (31.8% response rate). According to the survey, all geriatricians felt a shared responsibility to participate in research. However, 101/171 (59.1%) geriatricians did not participate. Of the 70 geriatricians that participated in research, 20 (28.6%) joined a research consortium. Following the survey, 21 semi-structured interviews were conducted. Lack of time, financial resources, and inadequate training during geriatric residency were identified as barriers to participate in research. Interviews revealed that earlier positive experiences with research and synergic collaboration with colleagues encourage participation of geriatricians in research.

**Discussion:**

More focus on research during geriatric medicine residency could foster long-term research involvement. This can be achieved by providing increased exposure to research, positive experiences with research, and access to skilled supervisors.

**Conclusion:**

Barriers and facilitators should be addressed in policies to promote greater involvement of geriatricians in research and research consortia.

**Supplementary Information:**

The online version contains supplementary material available at https://doi.org/10.1007/s40520-026-03426-z.

## Introduction

With the aging population, there is a growing need for research in geriatrics to drive knowledge development and improve patient care. Currently, the involvement of geriatricians in research is behind that of other specialties [1]. For instance, geriatricians are one-third less likely to obtain a PhD compared to other specialists in internal medicine in Norway [2]. Initiating and engaging in research within geriatrics is often challenging, particularly due to the limited number of clinicians, insufficient research infrastructure, and vulnerability of the patient group, which complicates their study participation [2, 3]. To address these challenges, the Geriatric Medicine Research Collaborative, a trainee-led collaboration in the UK that aims to improve access to and understanding of research, highlighted the need to increase the skills of geriatric medicine trainees in research methodology and more involvement of trainees in all phases of the research cycle [4].

In addition, pooling resources through collaboration in research consortia could offer a viable solution. A research consortium is a formalized collaboration that connects individuals or organisations to address a common set of questions or goals using a defined structure and governance model [5]. Research consortia also enable increased access to experts and larger sample sizes [6]. In addition, it allows collaborative decision making and better generalizability of the study findings [7].

Although research collaborations are promoted by governmental and funding organizations, the creation and operation of research consortia can be challenging. Challenges include issues related to operationalisation (planning, participation and relationship between members) and the structure of the consortium (management and leadership) [8, 9]. As an example, within geriatrics, the PreColo comparative effectiveness research consortium, which aimed to investigate the effectiveness of prehabilitation in older patients with colorectal cancer, also faced multiple challenges. These challenges included a lack of stakeholder involvement, and a limited implementation rate of the intervention, which ultimately led to an insufficient sample size [10].

During the COVID-19 pandemic, multiple scientific projects and research consortia were successfully created in the field of geriatrics both in the Netherlands, the United Kingdom and worldwide [11–17]. The lack of knowledge about COVID-19 created an urgent need to collaborate for knowledge development. The pandemic forced successful collaborations, which highlighted opportunities to improve research and the creation of research consortia in this field. By reflecting on this period of accelerated research collaborations, we can gain insight into the factors that enabled these successful developments and consider how they might facilitate future research collaborations. Therefore, this study aims to explore the perceptions, motivations, needs and experiences of geriatricians with research and research consortia in the Netherlands.

## Methods

### Study design

This study is a sequential explanatory mixed-methods study, which consists of a quantitative component (online survey) followed by a qualitative component (semi-structured in-depth interviews) [18, 19]. We chose a mixed-methods study design as the semi-structured interviews helped to gain an in-depth understanding of findings from the online survey. The STROBE (STrengthening the Reporting of OBservational studies in Epidemiology) standards were used for reporting the quantitative component and the COREQ (COnsolidated criteria for Reporting Qualitative research) for reporting the qualitative component [20, 21]. The medical ethics committees of the Erasmus Medical Center waived the necessity for formal approval of the study, since it involves healthcare professionals being asked about a non-medical-scientific topic and it does not involve patient data.

### Quantitative study component

#### Design, setting and study participants

In the Netherlands, consultant geriatricians can be ‘clinical geriatricians’ and ‘internist-geriatricians’. Both specialties work in general (both university and non-university) or non-university psychiatric hospitals (as opposed to elderly care physicians who work in nursing homes mostly) [22]. For the purpose of this article, they will all be referred to as geriatricians or participants. All geriatricians were invited by e-mail to participate in an anonymous online survey. The e-mail was sent to all members of the Nederlandse Vereniging voor Klinische Geriatrie (NVKG, the Dutch Geriatrics Society) and to all internist-geriatrician members of the Nederlandse Internisten Vereniging (NIV, the Dutch Society of Internal Medicine), as all geriatricians are a member of one of these societies. To increase the response rate, reminders were sent four and six weeks after the initial invitation to participate. Information on the survey was shared in the newsletters of the NVKG and the NIV.

#### Quantitative data collection

Participants could fill out the survey between March 2024 and May 2024 using the online electronic data capture (EDC) platform Castor [23]. The questions for this survey were partially based on the Healthy ALLiances (HALL) framework (Supplemental Fig. 1) [24]. The Dutch HALL framework addresses prerequisites for successful collaborations between primary care and public health, and could – as a proxy - provide insight into factors that could facilitate successful research collaborations in geriatrics [24]. In short, the framework states that there are three clusters of factors that can either hinder or facilitate the success of alliances: institutional factors, personal factors of participants in the alliance, and factors relating to the organisation of the alliance. The survey questions were developed based on these three clusters, in addition to a comprehensive literature review and expert meetings [24–28]. The survey questions addressing personal factors focused on individual characteristics of the geriatrician, and their motivation to potentially participate in research and research consortia. Additionally, questions on needs and potential experiences with regard to research and research consortia were addressed, if applicable. Questions related to research consortia were only asked in case a participant had experience within a research consortium. The questions regarding institutional factors focussed on the organisation of research at the geriatrics department level and the hospital-wide level. The questions on organisational factors included the organisation of research within research consortia. Additionally, the survey addressed the perceived involvement of the scientific societies (NVKG and the NIV) in research. No questions were mandatory, except for the questions on whether the geriatrician participated in research, and (if applicable) in a research consortium. The survey was pilot-tested with four geriatricians to ensure the clarity of the questions and the ease of understanding the survey format in CASTOR.

#### Quantitative data analyses

Due to the anonymity of the online survey, it was possible for geriatricians to access the survey multiple times, which could result in duplicate entries. Therefore, we decided to exclude surveys with less than 50% of the questions completed to avoid possible duplicates. In addition, we did an analysis for potential duplicate responses by identifying matching cases based on the following descriptive variables: sex, age, specialty (clinical geriatrician, internist-geriatrician or both), type of department (clinical geriatrician vs. internal medicine), number of colleagues in department, years of working experience, type of hospital (university vs. non-university) and participation in research and a research consortium. Completing the survey after the introductory information was considered as informed consent. The categorical data were presented as proportions and percentages. Means and standard deviations or median and interquartile ranges were presented for continuous variables depending on the distribution of the data. The data were analysed using IBM SPSS statistics version 28.0.

### Qualitative study component

#### Design, setting and study participants

Purposive sampling was used to select participants for the interviews. The source population consisted of all geriatricians working in the Netherlands, regardless of whether they had completed the questionnaire. In order to include a wide variety of participants, we selected participants based on their sex, participation in research, years of work experience as a geriatrician, and type of hospital they work in. The interviews were conducted between May 2024 and September 2024 and were analysed with a thematic content analysis.

#### Qualitative data collection

The interviews were conducted by two trained researchers (JM and ISM). Participants were offered a choice for a face-to-face or an online interview via Microsoft Teams (version 24257.205.3165.2029). Data collection continued until data saturation had been reached, i.e., when no new themes emerged from the interviews. This was determined through continuous evaluation of the data and discussion between the data collectors and study team.

The interview guide was based on the quantitative survey from this study and a literature search and included open-end questions allowing respondents to answer in their own words [24–28]. The guide included questions concerning: (1) geriatricians’ background, training and working environments, (2) the definition of research and the activities it includes, (3) potential participation in research and research consortia, (4) motivations to (not) participate in research, (5) potential availability of support within the geriatrics department and hospital-wide for participating in research, (6) the perceptions of the geriatrics department and hospital regarding research, and (7) the perceived involvement of the NVKG and NIV in research. The interview guide was pilot tested. Interviews were audio recorded with participants’ consent and transcribed verbatim. To maximize the reliability and integrity of the results, we performed a member check.

#### Qualitative data analyses

ATLAS-ti web (Version 9.2.0) was used to analyse the data. The development of codes was a stepwise iterative process. Two researchers (JM and ISM) independently coded the first three transcripts inductively using the principle of open coding. In regular meetings, JM, ISM, HP, YD and MF discussed emerging themes, leading to refinement of the codes and code groups. After consensus on the codes, JM and ISM independently coded the next seven transcripts, adding new codes if necessary, and compared the results. The remaining 11 transcripts were subsequently coded by JM. Based on the iterative process moving backwards and forwards between transcripts and evolving findings, main themes and key elements in the interviews were identified and discussed in the team, until data saturation was reached. Quotations were selected that illustrated the main themes.

## Results

### Survey

**Fig. 1:**
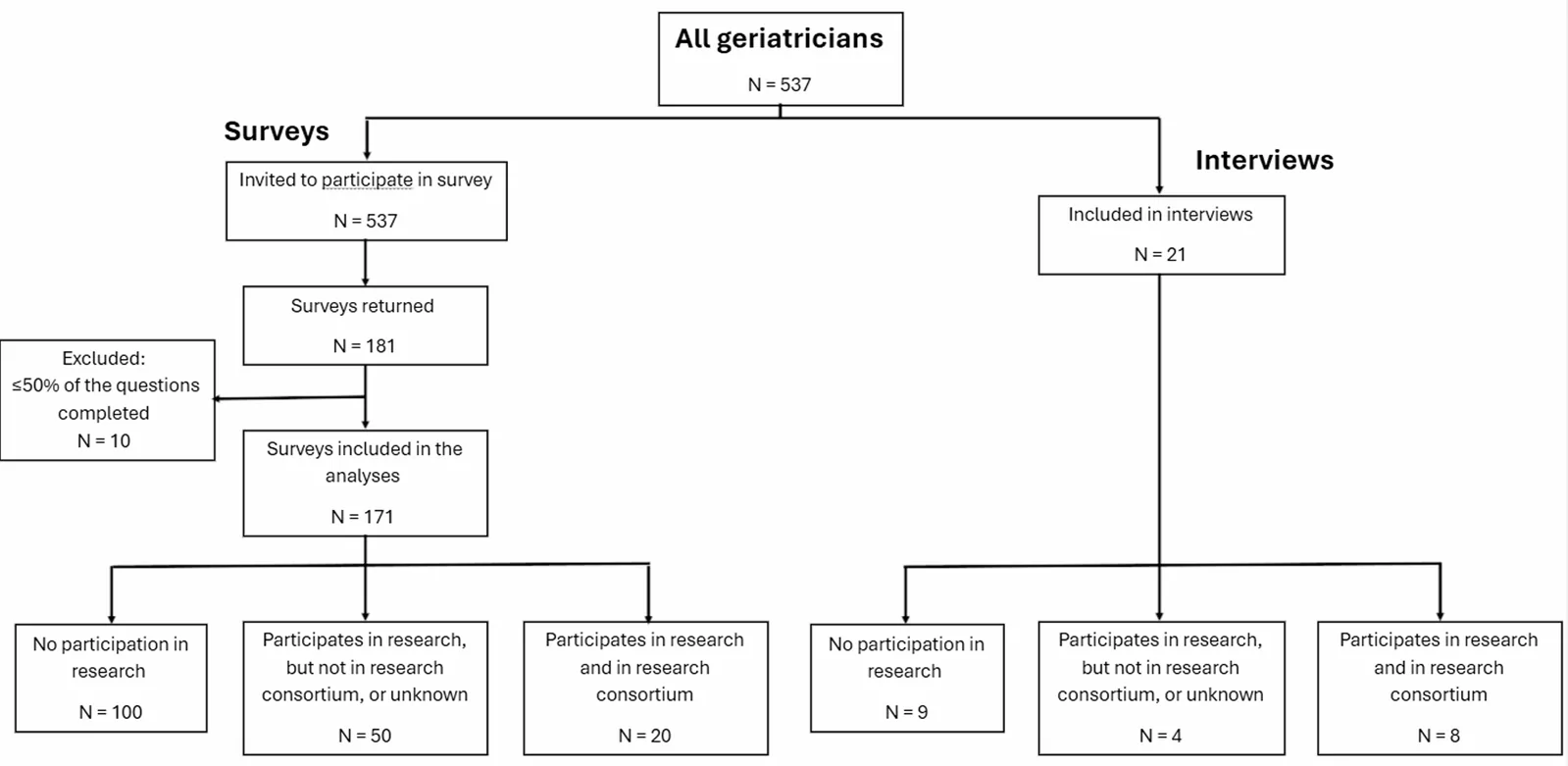
Flowchart of the study participants

A total of 537 geriatricians were invited to participate in the online survey. In total, 10 surveys with less than 50% of the questions completed were excluded. Two surveys were indicated as potential duplicates after additional analyses based on descriptive variables. However, since the responses to the open-ended questions differed, we decided to not classify them as duplicates. The surveys of 171 geriatricians were included in the analysis (response rate 31.8%), including 146 ‘clinical geriatricians’, 21 ‘internist-geriatricians’ and 4 who are both clinical geriatricians and internist-geriatricians (Tables 1 and Fig. 1). The median age of the study participants was 41 years (IQR = 37–50 years) and 137 (80.6%) were female. Most participants (59.1%) indicated that they did not participate in research. Of the 70 study participants who participated in research, 20 (28.6%) were also involved in a research consortium. The participants who were involved in a research consortium had a median working experience of 10 years (IQR: 7–19) years, compared to 7 years (IQR: 4–17) of those not working in a research consortium. Among participants holding a PhD, 13 (26.0%) were currently not involved in research. The geriatrics departments were staffed by a median of 7 (IQR: 6–9) geriatricians. Almost half of the participants (43.1%) indicated that dedicated time for research was available within their geriatrics department. More than three-quarters (76.9%) of the participants had access to a scientific office in their hospital. In total, 56.3% of the participants felt that the NVKG and NIV could do more to stimulate research.

**Table 1:** Characteristics of survey participants stratified by participation in research

	Number available *n* (%)	All geriatricians (*n* = 171; 100%)	No participation in research (*n* = 101; 59.1%)	Participates in research, but not in research consortium or unknown (*n* = 50; 29.2%)	Participates in research and in research consortium (*n* = 20; 11.7%)
**Individual**					
Female, n (%)	170 (99.4)	137 (80.6)	81 (81.0)	40 (80.0)	16 (80.0)
Age, median (IQR)	171 (100)	41 (37–50)	40 (36–47)	42 (37–50)	44 (39–53)
Years working as geriatrician, median (IQR)	159 (93.0)	7 (3–15)	6 (3–15)	7 (4–17)	10 (7–19)
Hospital, n (%)	160 (93.6)				
University hospital		20 (12.5)	1 (1.1)	12 (25.5)	7 (36.8)
Non-university hospital		131 (81.9)	87 (92.6)	33 (70.2)	11 (57.9)
University and non-university hospital		2 (1.3)	0 (0.0)	1 (2.1)	1 (5.3)
Psychiatric hospital		7 (4.4)	6 (6.4)	1 (2.1)	0 (0.0)
PhD, n (%)	155 (90.6)	50 (32.2)	13 (14.4)	20 (43.5)	17 (89.5)
**Geriatrics department**					
Number of geriatricians in staff, median (IQR)	157 (91.8)	7 (6–9)	7 (5–8)	7 (6–8)	8 (6–10)
Number of geriatricians with PhD, median (IQR)	154 (90.1)	1 (1–3)	1 (0–2)	2 (1–4)	3 (2–6)
Trains geriatric medicine trainees, n (%)	156 (91.2)	77 (49.4)	36 (37.9)	26 (60.5)	15 (83.3)
Participates in research, n (%)	156 (91.2)	111 (71.2)	52 (54.7)	41 (95.3)	18 (100.0)
Structural time for research, n (%)	153 (89.5)	66 (43.1)	29 (31.2)	25 (59.5)	12 (66.7)
Structural support ((human) resources) for research, n (%)	142 (83.0)	41 (28.9)	18 (21.2)	16 (40.0)	7 (41.2)
**Hospital**					
Scientific office present, n (%)	147 (86.0)	113 (76.9)	58 (65.9)	39 (92.9)	16 (94.1)
**NVKG (Dutch Geriatrics Society) and NIV (the Dutch Society for Internal Medicine)**					
Method of learning about new research within geriatrics, n (%)	171 (100)				
Newsletter from NVKG or NIV		129 (75.4)	78 (77.2)	36 (72.0)	15 (75.0)
Joint scientific committee		19 (11.1)	8 (7.9)	4 (8.0)	7 (35.0)
Verbally		71 (41.5)	39 (38.6)	20 (40.0)	12 (60.0)
Yearly scientific congress NVKG (Geriatriedagen)		112 (65.5)	76 (75.2)	28 (56.0)	8 (40.0)
Positively stimulated by the NVKG/NIV newsletter to participate in research within geriatrics, n (%)	113 (66.1)				
Not affected (at all)		62 (54.9)	42 (62.7)	17 (50.0)	3 (21.4)
Neutral		39 (34.5)	21 (31.3)	12 (35.3)	6 (42.9)
Affected (at all)		12 (10.6)	4 (6.0)	5 (14.7)	3 (21.4)
The NVKG/NIV should stimulate involvement in research more, n (%)	142 (83.0)	80 (56.3)	41 (48.2)	25 (62.5)	14 (82.4)

The characteristics of the participants stratified by type of hospital are presented in Supplemental Table 1. Participants working in university hospitals (13.8%) had significantly more years of work experience, with a median of 13 years (IQR: 6–20), compared with participants working in non-university hospitals (median: 6 years (IQR: 3–14)). Participation in research was higher among participants in university hospitals, with 95.5% participating, compared to 32.6% in non-university hospitals. In total, 82.4% of the participants in university hospitals reported having structural time for research, versus 38.6% of those in non-university hospitals.

Perceptions and motivations regarding participation in- and conducting research are presented in Table 2. In total, 70 participants indicated that they were involved in research. The amount of time spent on research varied, but most participants participating in research (67.1%) spent 1–5 h per week on research. More than half (67.7%) indicated they would like to devote more time to research. All participants participating in research felt a shared responsibility to engage in research. The main motivations for performing research were improving patient care (81.4%) and having an intellectual challenge (64.3%). The main reasons for the study participants to not participate in research were a lack of time (57.4%) and not feeling competent to perform research (38.6%). Collaboration with other researchers is important to half (50.0%) of the participants. Almost half (47.4%) of the participants reported that participation in research lowered the threshold for future participation in research. Of the participants who were involved in a research consortium, 95.0% reported that an intellectual challenge was the main motivation for performing research.

**Table 2:** Perceptions and motivations regarding participation in- and performing research

	Number available *n* (%)	All geriatricians participating in research (*n* = 70; 100%)	Participates in research, but not in a research consortium or unknown (*n* = 50; 71.4%)	Participates in research and in a research consortium (*n* = 20; 28.6%)
Time per week spent on research, *n* (%)	70 (100)			
1–5 h		47 (67.1)	40 (80.0)	7 (35.0)
6–10 h		19 (27.1)	10 (20.0)	9 (45.0)
> 10 h		4 (5.8)	0 (0.0)	4 (20.0)
Would like to spend more time on research, n (%)	65 (92.9)	44 (67.7)	27 (58.7)	17 (89.5)
Main motivations for participating in research, n (%)	70 (100)			
Building a network		14 (20.0)	7 (14.0)	7 (35.0)
Improve care for older adults		57 (81.4)	39 (78.0)	18 (90.0)
Changing activities		39 (55.7)	27 (54.0)	12 (60.0)
Intellectual challenge		45 (64.3)	26 (52.0)	19 (95.0)
Create scientific output		13 (18.6)	6 (12.0)	7 (35.0)
Type of research	70 (100)			
Nationwide studies		31 (44.3)	14 (28.0)	17 (85.0)
In the context of education geriatric medicine trainees		26 (37.1)	12 (24.0)	14 (70.0)
Research within own hospital		39 (55.7)	23 (46.0)	16 (80.0)
Contribution to research will lead to improvement of care in practice, n (%)	70 (100)			
I don’t have that feeling (at all)		10 (14.3)	9 (18.0)	1 (5.0)
Neutral		24 (34.3)	20 (40.0)	4 (20.0)
I have that feeling (a lot)		36 (51.5)	21 (42.0)	15 (75.0)
As a profession, we have a shared responsibility to participate in research, n (%)	69 (98.6)	69 (100)	49 (100)	20 (100)
I experience responsibility to contribute to research within geriatrics, n (%)	59 (84.3)			
No responsibility (at all)		11 (18.7)	11 (27.5)	0 (0.0)
Neutral		16 (27.0)	13 (32.5)	3 (15.8)
(A lot of) responsibility		32 (54.3)	16 (40.0)	16 (84.2)
Contributing to research gives me satisfaction, n (%)	62 (88.6)			
No satisfaction (at all)		9 (14.5)	9 (20.9)	0 (0.0)
Neutral		11 (17.7)	9 (20.9)	2 (10.5)
(A lot of) satisfaction		42 (67.8)	25 (58.2)	17 (89.5)
Participation in previous research lowered the threshold for future research, n (%)	59 (84.3)			
Not lowered (at all)		16 (27.1)	12 (29.3)	4 (22.2)
Neutral		15 (25.4)	11 (26.8)	4 (22.2)
Lowered (a lot)		28 (47.4)	18 (43.9)	10 (55.6)
Collaboration with other researchers is important to me, n (%)	60 (85.7)			
Not important (at all)		3 (5.0)	3 (7.3)	0 (0.0)
Neutral		15 (25.0)	14 (34.1)	1 (5.3)
(Very) important		42 (50.0)	24 (58.5)	18 (94.8)

The experiences of the study participants with research consortia and the organisation of research consortia are presented in Table 3. Most participants involved in a research consortium (*N* = 20) agreed that it is important that contributions are recognized by consortium members (55.0%). In total, 8 (40.0%) participants received recognition from their fellow consortium members. The majority of participants felt that the research consortia they were involved in were transparent regarding the agreements on authorships (57.9%), finances (68.6%) and data sharing (60.2%).

**Table 3:** Experiences in- and organization of research consortium

	Number available *n* (%)	Participates in research and in a research consortium (*n* = 20; 100%)
Important that contributions are recognized by consortium members, *n* (%)	20 (100)	
Not important (at all)		2 (10.0)
Neutral		7 (35.0)
(Very) important		11 (55.0)
I receive recognition for my contributions	20 (100)	
I don’t experience that (at all)		2 (10.0)
Neutral		10 (50.0)
I experience that (a lot)		8 (40.0)
Important that there are transparent agreements on authorship within a research consortium	19 (95.0)	
Not important (at all)		1 (5.3)
Neutral		1 (5.3)
(Very) important		17 (89.4)
I experience transparent agreements on authorship within a research consortium	19 (95.0)	
I don’t experience that (at all)		2 (10.5)
Neutral		6 (31.6)
I experience that (a lot)		11 (57.9)
Important that there are transparent agreements on data sharing within a research consortium	19 (95.0)	
Not important (at all)		0 (0.0)
Neutral		0 (0.0)
(Very) important		19 (100)
I experience transparent agreements on data sharing within a research consortium	19 (95.0)	
I don’t experience that (at all)		2 (10.5)
Neutral		5 (26.3)
I experience that (a lot)		12 (63.2)
Important that there are transparent agreements on financing within a research consortium	19 (95.0)	
Not important (at all)		0 (0.0)
Neutral		1 (5.2)
(Very) important		18 (94.8)
I experience transparent agreements on financing within a research consortium	19 (95.0)	
I don’t experience that (at all)		1 (5.3)
Neutral		5 (26.3)
I experience that (at all)		13 (68.4)

### Semi-structured interviews

We conducted 21 semi-structured interviews with Dutch geriatricians, of which 20 were conducted online and 1 face-to-face (Fig. 1). We included 5 geriatricians from a university hospital, 14 from a general non-university hospital, and 2 from a psychiatric non-university hospital (Table 4). The study participants had a median age of 41 years [IQR: 39–50] and a median of 9 years [IQR: 6–16] of experience in geriatrics. Of the 21 participants, 9 were not involved in research. Of the 12 participants involved in research, 8 were involved in a research consortium. The interviews lasted between 30 min and 66 min, with a mean of 46 min. Data analysis was performed according to 6 main themes: (1) perceptions of research, (2) motivations to initiate and participate in research, (3) needs for performing research, (4) collaboration within a research consortium, (5) views and support of the hospital on research, and (6) the perceived involvement of the scientific societies NVKG and the NIV in research. Illustrative quotes are listed in Table 5.

**Table 4:** Characteristics of geriatricians participating in interviews

	All geriatricians *n* = 21	No participation in research *n* = 9 (42.9%)	Participation in research *n* = 12 (57.1%)
Sex, Female, *n* (%)	17 (81.0)	6 (66.7)	11 (91.7)
Age, median [IQR]	41 [39–50]	41 [38–48]	45 [40–52]
Hospital, n (%)			
University hospital	5 (23.8)	1 (11.1)	4 (33.3)
Non-university hospital	14 (66.7)	7 (77.8)	7 (58.3)
Psychiatric hospital	2 (9.5)	1 (11.1)	1 (8.3)
Years of experience, median [IQR]	9 [6–16]	9 [6–12]	12 [5–20]
PhD, n (%)	11 (52.4)	0 (0.0)	11 (91.7)
Participation in a research consortium, n (%)	8 (38.1)	N.A.	8 (66.7)

**Table 5:** Qualitative findings related to research among geriatricians (*n* = 21)

Themes and codes	Illustrative quotes
1. **Perceptions about research**	
Personal characteristics and acquired skills	“I can delve into something endlessly. Once I start, I want to know how it is. But that fits with being a geriatrician; like Sherlock Holmes going through the literature first, seeing what is known, and then figuring it out for myself.” (Geriatrician 21, Psychiatric hospital – involved in research)
2. **Motivations to perform research**	
Study population	“Generally the interventions that we do in the older population are complex interventions, which requires a complicated approach. Developing and testing a complex intervention obviously involves a lot more steps than a single intervention, such as taking a pill. (Geriatrician 10, university hospital – involved in research)
Earlier experiences with research	“I had an internship where I didn’t have enough to do. So, I asked my supervisor, ‘Do you have something I can work on?’ She replied, ‘I’m curious about what we do with […] in our patients; we each handle it differently, and is that the right approach?’ I investigated it and presented the results. The response was very positive, and there was a follow-up question. That’s when I thought, ‘I want to find that out too.‘” (Geriatrician 4, non-university hospital, involved in research) “The experience that I have with scientific research… Everyone in geriatric medicine residency thought: we have to do this [scientific research], and it’s a lot of work and a hassle. We don’t really know what to do and we do not have somebody who can guide us. And you just had to do it on top of your regular work.” (Geriatrician 8, non-university hospital – not involved in research)
Geriatrics training within academia	“We are a new profession with relatively little academic history in the Netherlands, and therefore we receive relatively little scientific education during geriatric medicine residency. It is possible that you start and finish [your residency] completely in a non-university hospital, supervised by a geriatrician who does not have a PhD or who completed their PhD a long time ago and therefore cannot facilitate you in doing research. “ (Geriatrician 10, university hospital - involved in research)
Feeling responsible for conducting research	“We have decided within the department that you do at least one core task besides patient care [i.e. management, education or research]. So, there are Geriatricians who have the core task of scientific research, but others have other core tasks. We have chosen the tasks ourselves, depending on our interests and competencies.” (Geriatrician 2, university hospital – not involved in research)
Status	“What I saw was that many people conducting scientific research aimed to become a professor or wanted to make a name for themselves.” (Geriatrician 3, university hospital, involved in research)
Perceived clinical impact	“I conducted studies that, the next day in the consulting room or during discussions with a manager or colleague, made me think: I can already use these results. However, some studies have raised more awareness or reinforced the conclusion that we are doing the right thing.” (geriatrician 4, non-university hospital – involved in research)
3. **Barriers and facilitators for research**	
Time for research	“You cannot conduct scientific research and expect to do it only during working hours. You can, but then you have to work in a university hospital where you have a lot of dedicated time for it. It is not realistic in a non-university hospital. I think that’s what it is. It is not a big deal.” (Geriatrician 1, non-university hospital, involved in research) “When there is no time dedicated to research, I am not doing it. If you make it a priority, then it becomes normal. Right now, it is seen as extra. And it is a burden because you have to do it in your spare time somehow. Then it’s not much fun of course.” (Geriatrician 14, non-university hospital, not involved in research)
Support for conducting research	“When we ask hospitals to participate in research, we ask them to do a lot of work. We cannot easily extract data out of the electronic medical records. So, a lot of data have to be entered twice, and informed consent is often a lot of work. I think that we should be able to extract data out of the electronic medical records more easily and that informed consent should be easier to arrange in the Netherlands.” (Geriatrician 6, university hospital – involved in research) “What was also great was that, eventually, the research assistant from […] was able to help us with the data collection. That was a huge help because we would not have been able to do that ourselves. You simply cannot do it without funding. Being in a research consortium provides financial resources to arrange and share that kind of support for each other.” (Geriatrician 1, non-university hospital – involved in research)
Conflicting interests and regulations	“Submitting your study to the Medical Research Ethics Committee (MREC) requires an insane amount of time and energy. The MREC should be a lot easier for care evaluation studies. Although it is said that care evaluation studies should not be subject to MREC requirements, in practice they often are.” (Geriatrician 6, university hospital - involved in research)
4. **Research consortium**	
	“When I started doing this European project, I did not know anyone with experience in this field. I asked the neighbouring university hospital, but they said: we can only help you if we can be added to the project application. Then I said: no, that’s impossible, because it’s all formalized. Then, they would have had to take over from me [as head applicant]. So, there was not a lot of help available. (Geriatrician 4, non-university hospital – involved in research) “I find working with other researchers very inspiring; I work often with hospital pharmacists and with colleagues at the European level. That is very valuable, when you are with more people it always gets better.” (Geriatrician 13, university hospital – involved in research) “Sometimes you are not informed about the results [after including patients for a research consortium] and you miss, it sounds very childish, but some kind of credits and the ‘thank you’ or something. Sometimes a colleague has co-published, but not always”. (Geriatrician 20, non-university hospital – not involved in research)
5. **Views of non-university vs. university hospital regarding research**	
	“I cannot initiate scientific research without available resources; I cannot make something out of nothing. When I heard from the special interest group […] that the […]study was coming up, I just asked: can I participate? And I was allowed to.” (Geriatrician 9, non-university hospital – involved in research) “I think securing funding for research is a general problem. It demands a significant time investment, which is impossible within regular working hours. Not only geriatricians in non-university hospitals who do not have dedicated time for research struggle with this, but even those who have dedicated time face this challenge, because their workload increases accordingly.” (Geriatrician 10, university hospital – involved in research)
6. **Involvement of the scientific societies NVKG and NIV in research**	
	“I think that they could share their vision a bit more; that we all have to do scientific research and that we very much need the non-university hospitals, and that it should be a normal part of our work. And that they could also look at: how could we support this better by bringing hospitals that would very much like to do this into contact with centres where it is going well, to learn from each other.” (Geriatrician 6, university hospital – involved in research)

#### Perceptions about research

##### Definition of research and associated activities

Participants described research as a broad concept, but primarily as systematically answering a clinical research question with the aim of improving healthcare. They identified several components of research, including preparatory activities, executive activities, supervisory activities, knowledge dissemination, and facilitating activities. Most participants did not consider recruiting patients for involvement in research as participating in research. They felt they were not participating in research unless they were involved in aspects such as designing the study, contributing ideas, writing, or analysing data.

##### Personal characteristics and acquired skills

Several personal characteristics were mentioned as beneficial to participate in research, such as creativity, curiosity, a feeling of responsibility, and intrinsic motivation. In addition, acquired skills were mentioned, such as academic writing, an understanding of research methodology and statistics, but also an understanding of the dynamics and possible conflicts within a group of researchers.

#### Motivations to perform research

##### Emotions involved in research

Several participants found enjoyment and fulfilment in participating in research. Collaborating with colleagues was seen as motivating and rewarding, just like supervising scientific activities of geriatric medicine trainees. However, negative emotions were also expressed. A common sentiment was the difficulty managing both research and clinical duties simultaneously. In situations of staff shortages, the demands of patient care take priority over participating in research. Moreover, participants mentioned feeling guilty towards colleagues when prioritising research over patient care. To prevent conflicts within the geriatrics department, geriatricians emphasized the importance of keeping their colleagues informed about their research projects.

##### Study population

Participants noted that participating in research with geriatric patients is challenging as providing informed consent may be difficult due to lack of patients’ decisional capacity or intercurrent diseases. Therefore, research generally favour non-frail older adults or younger adults. In addition, geriatric patients can face challenges related to traveling and attending in-person hospital examinations with certain tests being potentially too demanding. As frail geriatric patients are often excluded from clinical trials, the study participants felt the obligation to focus especially on this population in their research.

##### Earlier experiences with research

The participants’ involvement in research was greatly influenced by their past experiences. Positive experiences with research during medical school and geriatric medicine residency could result in continued interest in research. Furthermore, having access to an engaged mentor with strong research skills who offered adequate supervision was an important factor in the geriatricians’ decision to continue research after completing their residency.

However, some participants mentioned that geriatricians who coach and supervise geriatric medicine trainees in their required research project are often not involved in research themselves and/or have not received adequate training in research. This limits their ability to effectively teach research skills to geriatric medicine trainees and supervise their required research project. In addition to a lack of positive exposure to research during medical school and geriatric medicine residency, negative personal experiences with research and discouraging stories from colleagues also prevent geriatricians from continuing research after residency. Those who did not participate in research often mentioned a lack of confidence in their research competences. This sense of inadequacy is viewed as barrier to eventually pursuing research.

##### Geriatrics training within academia

Participants thought that less research is conducted within geriatrics compared to other specialties. They attributed this mainly to the relatively small number of academics in the field and that geriatrics is a relative newer field compared to other specialties. During residency, geriatricians may work solely in non-university hospitals and may never have the opportunity to work with a geriatrician who holds a PhD or is actively engaged in research.

##### Feeling responsible to participate in research

Most participants felt a responsibility to contribute to research, especially with regards to improving patient care. Participants who were not involved in research also felt less responsibility to do so. However, these geriatricians did feel a responsibility to engage in other activities next to patient care, such as management or education. Participants emphasized the importance of dividing these activities equally among their colleagues.

##### Status

Research was perceived as a way of gaining status. This status could be individual or, for example, for a hospital wanting to maintain a ‘top clinical research’ status.

##### Perceived clinical impact

The perceived clinical impact of a study seemed to be important in the motivation to participate in research. Participants generally felt that the research they participated in had a significant clinical impact. However, some study participants, both with and without a PhD, noted that obtaining a PhD would not make them better medical doctors.

#### Barriers and facilitators for research

##### Time for research

Lack of time was identified as a limiting factor for participating in research. On average, only a few geriatricians in each geriatric department had dedicated time for research, and limited or no allocated time often forced them to conduct research during their spare time. Participants had varying opinions on this issue. For some, working outside of scheduled work hours was seen as a necessity to be able to participate in research, given the competing demands of their clinical responsibilities. Additionally, participants experienced that research required a varying time investment, making it challenging to balance it with clinical activities. Writing grant applications were also highlighted as a particular time-consuming aspect of research.

##### Support for participating in research

Most participants mentioned a lack of (human) resources for participating in research. They pointed out that data acquisition was especially challenging without additional support, such as research nurses or students. Accordingly, participants highlighted the importance of teamwork in participating in research. Many emphasize the need for collaboration within a multidisciplinary team, including epidemiologists or statisticians, which are often lacking in non-university hospitals. Additionally, they mentioned that their electronic medical records (EMR) were insufficiently equipped for (automatic) data extraction. There is a need to ensure that data can be more easily extracted and that informed consent is more efficiently regulated. To overcome the shortages in (human) resources, many geriatricians collaborate with colleagues from other hospitals, which helps them to compensate for the lack of support.

##### Conflicting interests and regulations

Several conflicting interests were at play within research, the main ones being finances and publications. Some participants mentioned that they focused mostly on studies which are not subject to the Medical Research Involving Human Subjects act, because they are relatively easy to conduct. In addition, the lengthy ethical and legal procedures for participating in scientific research were mentioned as demotivating.

#### Research consortium

The level of participation within a research consortium varied among participants. Some took on leadership roles or were participating in the steering committees, while others facilitated patient inclusion. Participants from non-university hospitals perceived it as challenging to take on leadership roles, since funding organizations often require a university hospital to be the head applicant for funding. Additionally, participants noted that the research questions that arise in non-university hospitals may differ from those in university hospitals, as they often see a different patient population, which are not always reflected in the national research agenda. As a potential solution, a participant suggested to foster regional collaboration by establishing a learning community. Additionally, investing in one’s personal network through site visits play an important role in improving collaboration between hospitals. Clear leadership was considered important in the organization of a research consortium. Geriatricians in a non-university hospital sometimes felt that they received too little feedback and rewards after contributing to a research consortium, for instance after including patients. Geriatricians did not mention problems with the division of authorships if it was properly documented beforehand.

#### Views and support of the hospital with regard to research

Geriatricians from non-university hospitals felt that geriatricians from university hospitals had more dedicated time and financial resources for research. They found that the focus in non-university hospitals was mainly on patient care, making it difficult to participate in research. To improve research, collaboration between university and non-university hospitals in research could be improved. Geriatricians explained that each hospital had a scientific office that could assist with research design and statistical analysis to enable them to contribute to research.

#### Involvement of the scientific societies NVKG and NIV in research

Most participants were aware of the involvement of the NVKG and NIV in research. Geriatricians were updated on upcoming and ongoing research through the newsletters of these societies. Geriatricians recommended having an overview of ongoing studies at each centre, which would make it clear which studies they could potentially get involved in. They also suggested reaching out to geriatricians in a more targeted and personal way, for instance through personal emails. Furthermore, geriatricians expressed a need for clear feedback regarding the national research agenda. It should be made evident who leads each study on the list and which opportunities exist for other geriatricians to join or contribute. Participants also indicate that guidance and an overview of available funding opportunities would be helpful. Having access to support when writing a grant application could potentially lower the barrier to participation in research.

## Discussion

This sequential explanatory mixed-methods study aimed to investigate the perceptions, motivations, needs and experiences of Dutch geriatricians with research and research consortia. The quantitative data showed that although the importance of research is recognised, more than half of the geriatricians do not participate in research. Qualitative data revealed that earlier positive experiences with research and synergic collaboration with colleagues stimulate research involvement, whereas barriers, such as lack of time and resources, and feelings of incompetence, hinder the involvement of geriatricians in research. Clear leadership, reward for and active participation of collaborating centers was seen as important for research consortia.

Geriatricians emphasised that previous experiences with research during medical school or the obligated research project during residency, play a significant role in the decision whether they continue participating in research as a medical specialist. Geriatricians with positive earlier experiences with research mentioned receiving good supervision by geriatricians who were well-trained and experienced in research. Having a supervisor who is actively involved in research provides essential guidance and support. Additionally, access to role models in the field further motivates geriatricians to engage in research and develop their research skills. A study from the Penn State College of Medicine, in which a survey was sent to classes who completed their medical training, mentioned that participation in research as a medical student is an important determinant for future involvement in research [29]. Additionally, earlier research noted that if geriatric medicine trainees do not develop sufficient expertise in research methodologies during their training, this could interfere with them getting involved in the training of the next generation of geriatricians [30]. Moreover, a meeting of UK academic geriatricians in 2018 recognised that support for early-career clinical researchers and good mentorship at the early stage of the career of a geriatrician is critical to foster long-term involvement in research. This highlights the importance of positive research experiences during geriatric medicine residency to foster long-term involvement of geriatricians in research. Geriatricians who did not continue research after their residency reported in the interviews that they felt unsure about their research capabilities, due to a lack of exposure to research or a lack of adequate supervision and guidance during their geriatric medicine residency. Geriatricians also highlighted that if they do not start with research immediately after their geriatric medicine residency, they feel it becomes increasingly difficult to get involved later in their career. Established responsibilities such as management or education take priority, leaving little time to maintain and develop the necessary research skills and build collaborations.

The main barrier for participating in research according to the survey was a lack of time, which is also mentioned in previous studies. A survey send out in 2015 to almost 2000 medical doctors in the UK observed that a lack of time and funding are the main barriers for not performing research [31]. Furthermore, in a survey send out to UK higher specialist trainees in geriatric medicine in 2019, a lack of dedicated time was also mentioned as the most significant barrier for engagement in research [3]. Therefore, they propose to provide geriatric medicine trainees with designated time for research in order to build up research skills and engagement with research. This might also be a good proposal for geriatric medicine trainee in the Netherlands.

To address the lack of time, geriatricians in non-university hospitals in our study suggested to collaborate with geriatricians in university hospitals in research consortia, allowing certain tasks to be delegated. However, the interviews disclosed several challenges in the collaboration between university and non-university hospitals. Geriatricians from non-university hospitals often did not feel involved enough in research consortia and felt that their contributions in e.g. patient inclusion were not valued enough by their academic colleagues. This finding aligns with an interview study investigating barriers for collaborative research in the primary care setting, which also observed that effective communication between centres is essential for multi-centre research [32]. To foster collaboration between university- and non-university hospitals within research consortia, organisations such as the NVKG and the NIV could develop a more comprehensive policy regarding collaboration between centres and create an overview of ongoing studies and opportunities to collaborate, both at the national and regional level. In our study, 71.4% of the geriatricians participated in research were not involved in a research consortium. This suggests that there is room for more collaboration in the Netherlands. Geriatricians emphasized that within a research consortium a dedicated research team is needed, including a statistician and/or epidemiologist.

Along with a lack of time for research, a lack of funding was also frequently mentioned by geriatricians as barrier to participate in research. Difficulties arose from the limited availability of funding opportunities in geriatrics, compared to other areas such as cancer or cardiovascular disease [2]. Collaborations with organ specialists to broaden the funding envelope might be essential for knowledge development and improving healthcare for older adults [1]. However, geriatricians may lack experience in the grant application process which may limit their chances of success.

Another barrier mentioned by geriatricians was the challenge of including older patients for research. Geriatric patients can face challenges related to travel and attending in-person hospital examinations, with certain tests being potentially too burdensome for this population. Difficulties with patient inclusion were described as demotivating. The challenges of inclusion of this specific patient population in research are recognized in the literature [1]. To overcome this challenge, the NVKG developed ‘guidelines for medical scientific research among older adults’ [33].

This study has several strengths including the mixed-methods approach, which allowed us to collect data using different methodologies. The survey allowed for quick and standardized data collection on a large variety of topics, whereas the semi-structured interviews added in-depth insights, allowing the geriatricians to elaborate on their perspectives and views in a more nuanced and detailed way. The survey is filled out by approximately one third of the Dutch Geriatricians (31.8%), representing a substantial proportion of the geriatricians in the Netherlands. In addition, to our knowledge, this is the first mixed-methods study exploring the perceptions, motivations, needs and experiences of geriatricians regarding research and research consortia.

Our study also has several limitations that need to be considered while interpreting the study results. The survey may be filled out by geriatricians who have a greater interest in research, potentially leading to an overrepresentation of their views (sampling bias). In addition, some responses are inherently shaped by current research participation. However, by using a mixed-methods approach we aimed to elaborate on the findings of the survey. In the semi-structured interviews, we tried to overcome this limitation by purposively sampling a mix of geriatricians who did and did not participate in research. Further, both the survey and the semi-structured interviews are sensitive to response bias; geriatricians could have provided more socially desirable answers, which do not reflect their actual views on a topic. However, the anonymity of the survey might have encouraged geriatricians to express their opinions freely. As the survey and interviews asked questions about research consortia, this might have directed participants’ attention towards this type of collaboration and might introduce positive bias towards research consortia. However, questions regarding research consortia were only asked to participants who participate(d) in a research consortium. Moreover, the challenges faced by Dutch hospital geriatricians in engaging with research are not unique to the Netherlands; they are shaped by hospital geriatricians in many other countries. In particular, the limited time allocated for research, insufficient funding, and the minimal exposure to research during the geriatric medicine residency are issues that also arise beyond the Dutch context. By addressing these barriers and highlighting potential solutions, our findings contribute to the broader challenge on strengthening research participation among hospital geriatricians.

To conclude, the results of this study indicate that more emphasis on research during the geriatric medicine residency might be beneficial to foster long-term research involvement. The increased exposure to research, positive experience with research and access to a skilled supervisor could play an important role in giving geriatricians the skills and competences they need to continue with research throughout their career. Furthermore, the findings suggest that joining studies of colleagues through research consortia could lower the threshold for participation in research, indicating that consortia may play an important role in increasing research involvement among geriatricians. The results of this study may provide guidance to promote greater involvement of geriatricians in research and research consortia, while also enhancing their scientific skills and expertise.

## Supplementary Information

Below is the link to the electronic supplementary material.

Supplementary Material 1

## Data Availability

The data that support the findings of this study are available from the corresponding author upon reasonable request.
