# Supplementary material for: Perceptions, motivations, needs and experiences of geriatricians with regard to research and research consortia
Source: Aging Clin Exp Res. 2026 Jun 29;38(1):192. doi: 10.1007/s40520-026-03426-z (PMC13582043; doi:10.1007/s40520-026-03426-z)
Supplement: Supplementary file 1 [file 40520_2026_3426_MOESM1_ESM.docx]

**Supplemental Figure 1.** The Healthy ALLiances (HALL) framework

**Supplemental Table 1.** Characteristics of the participants in the online survey stratified by type of hospital

|  | Number available  n (%) | All geriatricians  (n=160; 100%) | Non-university hospital (including psychiatric hospital)  (n=138; 86.3%) | University hospital  (n=22; 13.8%) |
| --- | --- | --- | --- | --- |
| **Individual** |  |  |  |  |
| Woman, n (%) | 159 (99.4) | 126 (79.2) | 110 (80.3) | 16 (72.7) |
| Age, median (IQR) | 160 (100) | 41 [37 – 48] | 40 [37 – 47] | 45 [39 – 53] |
| Years working as geriatrician, median (IQR) | 157 (98.1) | 7 [3 – 15] | 6 [3 – 14] | 13 [6 – 20] |
| Participation in SR, n (%) | 160 (100) |  |  |  |
| No participation in SR |  | 94 (58.8) | 93 (67.4) | 1 (4.5) |
| Participates in SR, but not in a research consortium or unknown |  | 47 (29.4) | 34 (24.6) | 13 (59.1) |
| Participates in SR and in a research consortium |  | 19 (11.9) | 11 (8.0) | 8 (36.4) |
| PhD, n (%) | 155 (96.9) | 50 (32.3) | 34 (25.6) | 16 (72.7) |
| **Geriatrics department** |  |  |  |  |
| Number of geriatricians in staff, median (IQR) | 149 (93.1) | 7 [6 – 9] | 7 (6 – 9) | 7 (6 – 10) |
| Number of geriatricians with PhD, median (IQR) | 146 (91.3) | 1 [1 – 3] | 1 (0 – 2) | 6 (5 – 8) |
| Educates geriatricians in-training, n (%) | 147 (91.9) | 72 (49.0) | 55 (42.3) | 22 (100) |
| Conducts SR, n (%) | 147 (91.9) | 104 (70.7) | 87 (66.9) | 17 (100) |
| Structural time for SR, n (%) | 144 (90.0) | 63 (43.8) | 49 (38.6) | 14 (82.4) |
| Has structural support, in the form of people and resources, for conducting SR, n (%) | 133 (83.1) | 39 (29.3) | 25 (21.6) | 14 (82.4) |
| **Hospital** |  |  |  |  |
| Scientific office present, n (%) | 138 (86.3) | 107 (77.5) | 93 (75.6) | 14 (93.3) |
| **Dutch Geriatrics Society (DGS) and the Dutch Society for Internal Medicine (DSIM)** | | | | |
| Member of the joint scientific committee of the DGS and the DSIM, n (%) | 139 (86.9) | 17 (12.2) | 12 (9.8) | 5 (31.3) |
| Way of learning about new SR within geriatrics, n (%) |  |  |  |  |
| Newsletter from DGS or DSIM | 160 (100) | 120 (75.0) | 110 (79.7) | 10 (45.5) |
| Joint scientific committee | 160 (100) | 17 (10.6) | 11 (8.0) | 6 (27.3) |
| Verbally | 160 (100) | 66 (41.3) | 58 (42.0) | 8 (36.4) |
| Yearly congress DGS (Geriatriedagen) | 160 (100) | 103 (64.4) | 95 (68.8) | 8 (36.4) |
| Positively affected by the DGS/DSIM newsletter to participate in SR within geriatrics, n (%) | 106 (66.3) |  |  |  |
| Not affected at all |  | 33 (31.1) | 32 (34.0) | 1 (8.3) |
| Not affected |  | 23 (21.7) | 20 (21.3) | 3 (25.0) |
| Neutral |  | 38 (35.8) | 32 (34.0) | 6 (50.0) |
| Affected |  | 10 (9.4) | 9 (9.6) | 1 (8.3) |
| Affected a lot |  | 2 (1.2) | 1 (1.1) | 1 (8.3) |
| The DGS/DSIM should encourage more to perform SR, n (%) | 133 (83.1) | 75 (56.4) | 65 (54.6) | 10 (71.4) |
